# Physical Pretreatments Applied in Three Commercial Kits for the Extraction of High-Quality DNA from Activated Sewage Sludge

**DOI:** 10.3390/ijms242015243

**Published:** 2023-10-17

**Authors:** Claudio Vásquez, Benjamín Leyton-Carcaman, Fernanda P. Cid-Alda, Iñaky Segovia, Fernanda Pinto, Michel Abanto

**Affiliations:** 1 Doctorado en Ciencias Mención Biología Celular y Molecular Aplicada, Universidad de La Frontera, Temuco 4811230, Chile; claudio.vasquez@ufrontera.cl (C.V.); benjamin.leyton@ufrontera.cl (B.L.-C.); 2 Scientific and Technological Bioresource Nucleus (BIOREN), Universidad de La Frontera, Temuco 4811230, Chile; 3 Carrera de Tecnología Médica, Facultad de Medicina, Universidad de La Frontera, Temuco 4811230, Chile; i.segovia01@ufromail.cl; 4 Departamento de Procesos Industriales, Facultad de Ingeniería, Universidad Católica de Temuco, Casilla 15-D, Temuco 4780000, Chile; fpinto@uct.cl

**Keywords:** DNA extraction, complex samples, next-generation sequencing, metagenomics, microbiome, bioreactor

## Abstract

Obtaining sufficient and high-quality genomic DNA from sludge samples is a fundamental issue of feasibility and comparability in genomic studies of microbial diversity. Commercial kits for soil are often used for the extraction of gDNA from sludge samples due to the lack of specific kits. However, the evaluation of the performance of commercial kits for sludge DNA extraction is scarce and optimization of these methods to obtain a high quantity and quality of DNA is necessary, especially for downstream genomic sequencing. Sequential batch reactors (SBRs) loaded with lignocellulosic biomass are used for the synthesis of renewable resources such as levulinic acid (LA), adipic acid (AA), and polyhydroxyalkanoates (PHAs), and the biochemical synthesis of these compounds is conducted through the inoculation of microbes present in the residual activated sludge (AS) obtained from a municipal wastewater treatment plant. To characterize these microbes, the extraction of DNA from residual sewage sludge was conducted with three different commercial kits: Nucleospin^®^ Soil from Macherey-Nagel, DNEasy^®^ PowerSoil^®^ from Qiagen, and E.Z.N.A.^®^ Plant DNA Kit from Omega BIO-TEK. Nevertheless, to obtain the highest load and quality of DNA for next-generation sequencing (NGS) analysis, different pretreatments and different combinations of these pretreatments were used. The pretreatments considered were an ultrasonic bath and a temperature of 80 °C, together and separately with different incubation time periods of 30, 60, and 90 min. The results obtained suggest a significant improvement in the efficiency and quality of DNA extraction with the three commercial extraction kits when used together with the ultrasonic bath and 80 °C for 60 min. Here, we were able to prove that physical pretreatments are a viable alternative to chemical lysis for DNA extraction from complex samples such as sludge.

## 1. Introduction

Feedstocks such as lignocellulosic biomass could be used to produce high-added value compounds such as levulinic acid (LA), adipic acid (AA), and polyhydroxyalkanoates (PHAs) moving from an economy based on fossil fuels, to an economy based on renewable resources [1,2]. For example, LAs as raw material, could be applied as fuel additives, antifreeze, textiles, animal feed, herbicides, pharmaceuticals, and flavor substances [3,4]. On the other hand, AA is a straight-chain dicarboxylic acid with many uses in chemical, pharmaceutical, and lubricant manufacturing, mainly for resins and nylon 6–6 fibers production [5,6]. Instead, PHAs are used as a bioplastic with physicochemical and mechanical properties like those in petroleum-based plastics and could also be used in medical, pharmaceutical, and food applications [7]. Sequential batch reactors (SBRs) are used for the conversion of lignocellulosic waste to LA, AA, and PHAs, and microbes from activated sludge (AS) from a municipal sludge wastewater treatment plant were used for the biochemical synthesis of these high-added value compounds [8,9]. Microbes from the AS develop mixed microbial cultures responsible for the conversion of different carbon sources from lignocellulosic waste such as synthetic, molasses, sugarcane bagasse, and grape pomace hydrolysate [10]. Nevertheless, these microbes are likely an unknown black box, and this is why it is important to determine the identity of these microbes.

Nowadays, next-generation sequencing (NGS) studies are conducted to identify these microorganisms usually described as a taxonomically and metabolically diverse microbial community with complex relationships among their members [11]. The main problem, however, is that the disruption of this activated sludge from SBRs is difficult to achieve and time-consuming due to a heavy encapsulation of microorganisms in flocs or aerobic granules [12,13,14]. These microbial populations are usually aggregated or embedded in a polymeric matrix or gel, referred to as extracellular polymeric substances (EPSs), commonly hard to disaggregate [13].

Commercial DNA extraction kits provide the protocols for the extraction of nucleic acids from standard samples, but the extraction of genomic DNA (gDNA) from SBR samples is challenging, especially in terms of achieving sufficient concentration for NGS analysis and identification of these microbes. For Illumina sequencing, a load above 5 ng·µL^−1^ is required as described by the protocol “16S Metagenomic Sequencing Library Preparation” (Illumina, Inc., San Diego, CA, USA) [15]. Whereas for third-generation sequencing such as Pacific Bioscience (PacBio) and Oxford Nanopore sequencing (ONS), related to long-length reads, even more than 10 times this amount of DNA is necessary and has been described as the main bottleneck for genome sequencing [16]. A high quantity and quality of DNA, beside the amplification of a certain marker gene, such as *16S rRNA gene* or internal transcribed spacer (ITS), allows the application of this gDNA extraction for whole genome sequencing approaches (WGS) and obtains a more comprehensive and major resolution of taxonomic and functional profiles.

In addition, a high quality of DNA free of contamination is necessary with 260/280 absorbances in the range of 1.7 and 2.0 [17]. Thus, commercial kits require additional steps to obtain a sufficient concentration of gDNA free of contamination. Vortexing with glass beads, for example, has been used for mechanical sludge lysis to allow better hydration with the extraction buffers [14], whereas CTAB was added to improve chemical lysis and help to precipitate polysaccharides and humic substances [18].

Here, we aimed to explore the gDNA extraction efficiency obtained with three commercial extraction kits combined with different physical pretreatments tested to determine their efficiency. The commercial kits used were Genomic DNA from Soil (Nucleospin^®^ Soil from Macherey-Nagel), DNEasy^®^ PowerSoil^®^ from Qiagen, and E.Z.N.A.^®^ Plant DNA Kit from Omega BIO-TEK, and the pretreatments tested here were an ultrasonic bath (UB) for 90 min, a temperature of 80 °C for 90 min and the combination of UB + 80 °C for different periods of 30, 60, and 90 min.

## 2. Results

**Figure 1:**
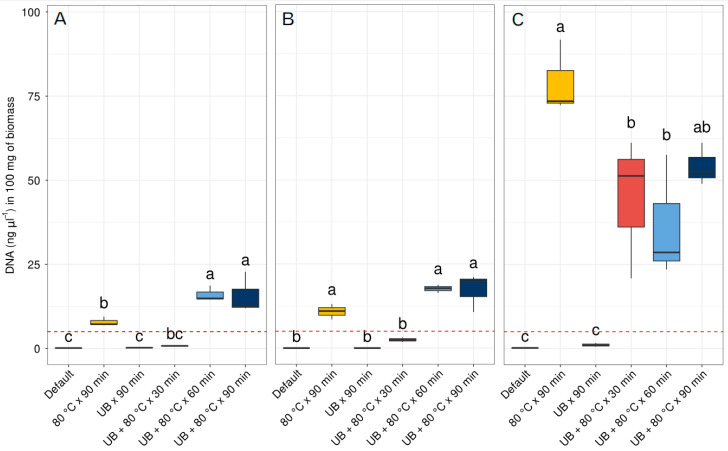
Concentration of DNA with the three commercial kits used. (**A**) Nucleospin^®^ Soil, (**B**) DNEasy^®^ PowerSoil^®^, and (**C**) E.Z.N.A.^®^ Plant DNA Kit. Different letters indicate statistical differences according to the results of ANOVA followed by Tukey’s post hoc test. UB = ultrasonic bath. The dashed red line indicates the optimal DNA concentration threshold for next-generation sequencing studies of 5 ng·µL^−1^.

The DNA concentrations acquired using the three available commercial kits as per the provider’s standard protocol were either extremely low or indetectable (<0.1 ng·µL^−1^) (Figure 1, Tables S1–S3). Notably, the application of 80 °C for 90 min and the combination of an ultrasonic bath (UB) with a temperature of 80 °C for 60 and 90 min prolonged periods resulted in increased DNA concentrations exceeding the mandatory 5 ng·µL^−1^ limit required for NGS across all three kits (as depicted in Figure 1). In the case of Nucleospin^®^ Soil in Figure 1A, it is possible to observe that the application of a temperature of 80 °C for 90 min is the main variable affecting DNA obtention, compared to the UB applied for 90 min with no significant differences from the default extraction. Similarly, the combined effect of UB + 80 °C for 30 min did not present significant differences from the default kit extraction protocol. The combined effect of 80 °C + UB for longer periods of 60 and 90 min were significantly higher than the default extraction protocol and surpasses the threshold concentration of 5 ng·µL^−1^ of DNA. In the case of the DNEasy^®^ PowerSoil^®^ kit, a similar trend to the Nucleospin^®^ Soil kit was observed (Figure 1A,B). The pretreatment of 80 °C for 90 min and the combined effect of UB + 80 °C temperature exposure for longer periods of 60 and 90 min were crucial to obtaining significant differences from the standard extraction protocol and a higher concentration above the threshold of 5 ng·µL^−1^ of gDNA.

Regarding the E.Z.N.A.^®^ Plant DNA Kit, as shown in Figure 1C, the application of 80 °C temperature was the main factor that significantly improved the DNA concentration, compared to the UB applied for 90 min, which did not show significant differences from the standard extraction protocol. As for the combined use of 80 °C + UB for the three periods of 30, 60, and 90 min, they resulted in significantly higher amounts of gDNA obtained with the standard kit extraction protocol. As can be seen in this figure, the differences in the concentration of gDNA obtained with the 80 °C applied for 90 min and the combination of 80 °C + UB for 30, 60, and 90 min were up to four times higher than the differences seen with the other two kits: Nucleospin^®^ Soil and the DNEasy^®^ PowerSoil^®^.

**Figure 2:**
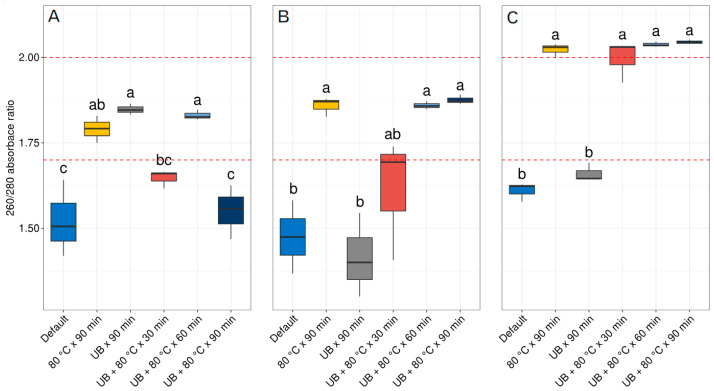
Quality of DNA extracted using three commercial kits. (**A**) Nucleospin^®^ Soil, (**B**) DNEasy^®^ PowerSoil^®^ and (**C**) E.Z.N.A.^®^ Plant DNA Kit, and the pretreatments applied before the extractions with the respective kits. Different letters indicate statistical differences according to the results of ANOVA followed by Tukey’s post hoc test. UB = ultrasonic bath. The dashed red line indicates the DNA quality threshold for the 260/280 ratio considered adequate in the ranges 1.7 and 2.0.

For the quality of the DNA, the 260/280 absorbance ratio was measured for the default extraction and all pretreatments tested. As shown in Figure 2A for the Nucleospin^®^ Soil kit, the application of 80 °C for 90 min, the UB applied for 90 min and the combination of UB + 80 °C for 60 min reached values in the ranges of 1.7 and 2.0 in the 260/280 absorbance ratio and were significantly higher than the default extraction protocol. The other pretreatments such as the combination of UB + 80 °C for 30 min, presented a significant difference from the default extraction; nevertheless, the values did not reach the quality ranges of 1.7 and 2.0 in the 260/280 absorbance ratio. Whereas with the pretreatment combination of UB + 80 °C for 90 min, no statistical differences were seen from the default extraction protocol and presented an average value of 1550, much lower than the ranges of 1.7 and 2.0 accepted for the 260/280 absorbance ratio (Table S1). Regarding the DNEasy^®^ PowerSoil^®^ kit, as seen in Figure 2B, qualities with significant differences from the default extraction protocol were seen when a temperature of 80 °C was applied for 90 min, and the combination of UB + 80 °C for 60 and 90 min reached the threshold for quality between 1.7 and 2.0 in the 260/280 absorbance ratio. Using this kit, the combination of UB + 80 °C for 30 min presented a significant difference with the default extraction protocol; however, the average did not reach the quality threshold of 1.7 and 2.0 in the 260/280 absorbance ratio. Regarding the application of UB for 90 min alone, no statistical difference was obtained compared to the default extraction protocol. Finally, for the E.Z.N.A.^®^ Plant DNA Kit, the application of 80 °C for 90 min and the combination of UB + 80 °C temperature no matter the time periods used presented a significant difference from the default extraction protocol, surpassing the quality threshold ranges of 1.7 and 2.0 in the 260/280 absorbance ratio. The pretreatment of UB for 90 min instead did not present any significant difference from the default extraction protocol and did not reach the threshold ranges for the 260/280 absorbance ratio.

Regarding the time required for each protocol, the Nucleospin^®^ Soil has a standard extraction time of approximately 25 min per sample. The DNEasy^®^ PowerSoil^®^ extraction protocol requires approximately 30 min per sample and the E.Z.N.A.^®^ Plant DNA Kit requires approximately 40 min per sample. For the pretreatments tested, applications of 80 °C for 90 min improved the quantity and quality of DNA, but the combination of UB + 80 °C helped to reduce the time from 1.5 h to 1 h and even half an hour in the case of the E.Z.N.A.^®^ Plant DNA Kit.

## 3. Discussion

As shown in the results, we could observe here that the pretreatments tested were essential to improve the quantity and quality of gDNA extractions from SBR samples for NGS and effectively favored the amounts of gDNA obtained with the three commercial kits used. Although the three kits responded adequately to the pretreatments tested, the kit that obtained the highest concentrations of gDNA was the E.Z.N.A.^®^ Plant DNA Kit with the pretreatments of 80 °C for 90 min and the combination of UB + 80 °C for 30, 60, and 90 min. This was followed by the two other soil extraction kits, Nucleospin^®^ Soil and DNEasy^®^ PowerSoil^®^, which showed a similar response to the pretreatments with higher DNA concentrations and quality with the pre-treatments of 80 °C for 90 min and the combination of UB + 80 °C for 60 min.

One of the reasons that could explain a better performance of the commercial kit for plants, is that it includes reagents to break down the rigid polysaccharide cell wall and other compounds such as secondary metabolites like phenols, carbohydrates, and waxes that protect plant cells and hamper the extraction of DNA [14,19]. These E.Z.N.A.^®^ Plant DNA Kit reagents, in the presence of sonication and high temperature, probably helped in the disruption of the EPS that hampered the obtention of DNA from the SBRs.

Regarding the 260/280 absorbance ratio, the quality obtained with the E.Z.N.A.^®^ Plant DNA Kit was on average above the threshold of 1.7 and 2.0 for the 260/280 absorbance ratio established by Chen et al. (2010) [17]. In the same line, other studies established that the accepted absorbances could vary between 1.6 and 1.9 [20,21]. In general, the peak of UV absorption occurs at 260 nm for DNA and at 280 nm for proteins, and a 260/280 absorbance ratio of 1.8 indicates that the extraction is free from protein contamination [14,17,22]. Nevertheless, higher values slightly above 2.0 also indicate that the sample contains more DNA than proteins in the extraction obtained. Indeed, other studies, such as that of Vilanova et al., 2020 [23], accepted values slightly above 2.0 due to the high concentration of DNA and the effective results obtained by enzymatic digestion.

It is important to note that other measurements not considered in this study, such as the 260/230 absorbance ratio [19,22,23] or absorbance at 340 nm [24,25], help to determine the presence of contaminants such as humic acids in the extracted gDNA. Therefore, it is important to consider this kind of measurement with similar samples in future studies. The main reason for considering this measurement is that humic acids from organic matter have similar size and charge of DNA resulting in their co-extraction and influencing the efficiency and purity of the extraction process [26,27]. Some of the mechanisms proposed in the literature to mitigate the effect of humic acids when present in gDNA extraction procedures include the use of aluminum sulphate and powdered activated charcoal [25,26]. Although the 260/230 absorbance ratio measurement was not considered in this study, the improvements in gDNA extraction obtained here were sufficient for the amplification of the *16S rRNA gene* and internal transcribed spacer (ITS) fragments for Illumina sequencing, which will be part of another manuscript in progress.

As mentioned above, the other two extraction kits responded similarly, with the pretreatments tested reaching the threshold concentration of 5 ng·µL^−1^ required for NGS when exposed to a temperature of 80 °C for 90 min and the combination of UB + 80 °C for 60 and 90 min. Regarding the quality obtained, it is important to mention that the only protocol that exhibited a high quantity of gDNA but a low quality, was the combination of UB + 80 °C for 90 min pretreatment with the kit Nucleospin^®^ Soil (Figure 2A). The main reason for the low quality obtained is probably that the longer time-lapsed exposure of 90 min favored not only DNA release but also a higher proportion of proteins from the EPSs.

As seen in our study, the mechanical effect of the UB for 90 min pretreatment was not sufficient to obtain significant differences with the standard extraction protocol with the three commercial kits tested. Similar results were obtained by Bourrain et al. (1999) [28] in which the sample dispersion was performed with an ultrasonic bath and by stirring in a cation exchange resin, with better results with the latter. Nevertheless, the use of a UB combined with a temperature of 80 °C helped to reduce the time from 90 min to 1 h (Figure 1 and Figure 2). In other studies, such as that of Shan et al. (2008) [22], the use of SDS, mechanical milling, and thermal shock were the most effective ways to obtain better DNA yield and quality.

The results obtained here show that the main variable that significantly improved gDNA extraction from SBR samples was the application of a temperature of 80 °C for 90 min alone and the combination of a temperature of 80 °C and a UB for 60 min. Previous studies have reported the use of a combination of mechanical and chemical lysis. For example, glass bead vortexing was used for mechanical sludge lysis to allow better hydration with the extraction buffers [14], while CTAB was added to improve chemical lysis and helped to precipitate polysaccharides and humic substances [18].

## 4. Materials and Methods

A residual sewage sludge sample was first homogenized with vortex for 2 min and aliquoted in 54 tubes of 1 mL to consider the three extraction kits, the six treatments of temperature and sonication added, and the three replicates per sample analyzed (3 × 6 × 3 = 54). The tubes were centrifuged for 10 min at 10,000× *g*, and the supernatant was removed. The resulting biomass was weighed, and biomasses were equalized to 100 mg.

Three commercial DNA extraction kits were used: Genomic DNA from Soil (Nucleospin^®^ Soil) Macherey-Nagel, Düren, Germany; DNEasy^®^ PowerSoil^®^ from Qiagen, Hilden, Germany; and E.Z.N.A.^®^ Plant DNA Kit from Omega BIO-TEK, Norcross, GA, USA. To maintain the initial conditions used with the kits Genomic DNA from Soil and the DNEasy^®^ PowerSoil^®^ which contain 2 mm steel beads to homogenize samples, this step was also added for the E.Z.N.A.^®^ Plant DNA Kit to homogenize samples. For the use of the three commercial kits, samples were homogenized twice in FastPrep-24^TM^ MP Biomedicals™ equipment, at 6.5 m s^−1^ per minute.

To improve the efficiency and quality of the DNA extractions, different pretreatments were tested: (1) the default extraction protocol described in each kit, (2) the application of a temperature of 80 °C in a dry bath incubator (Allsheng, model MK 200-1, Hangzhou, China) for 90 min, (3) a pretreatment with only an ultrasonic bath (UB) (Elmasonic E30H, Singen, Germany) for 90 min, (4) a UB combined with 80 °C for 30 min, (5) a UB combined with 80 °C for 60 min, and (6) a UB combined with 80 °C for 90 min. For each pretreatment, the extractions were conducted in triplicate and the median of concentrations measured were used for graph constructions and the concentrations were normalized to 100 mg of biomass. After the application of the different pretreatments tested, the extraction protocols were followed as described by the corresponding provider protocols.

The concentration of DNA from the samples extracted was found with equipment Qubit 4.0 (Invitrogen, Woodlands, Singapore) with a 1X dsDNA HS Assay Kit, following the manufacturer’s instructions. To determine the quality of the DNA obtained, the relation 260/280 absorbance was obtained with the spectrophotometer Synergy H1 Hybrid Reader (Biotek, Santa Clara, CA, USA), with an adaptor for the quantification of nucleic acids Take3 and each sample was read in duplicate following the manufacturer’s instructions. A load above 5 ng·µL^−1^ was established for Illumina sequencing as described by the protocol “16S Metagenomic Sequencing Library Preparation” (Illumina, Inc., San Diego, CA, USA) [16]. While the ranges of quality were established between 1.7 and 2.0 for the absorbance ratio of 260/280 as described by Chen et al. (2010) [17].

The visualization and statistical analysis were conducted In R language using the libraries from *ggpubr*, *ggplot2*, *car*, *agricolae*, and *dplyr* [29,30,31,32]. To evaluate the normal distribution of data, the Levene and Shapiro–Wilk test were first conducted, and to compare the pretreatments tested, a one-way ANOVA was conducted followed by a Tukey’s post hoc test to determine the significance of the results obtained.

## 5. Conclusions

As seen in this study, the pretreatments tested provided substantial improvement to the quantity and quality of genomic DNA (gDNA) from samples from sequential batch reactors’ (SBRs) residues extracted with commercial kits based on silica matrices where gDNA binds. For the three commercial kits tested, these pretreatments increased the concentration of high molecular weight gDNA and its quality, especially when a temperature of 80 °C was applied for 90 min, and in the treatments including a combination of 80 °C temperature and an ultrasonic bath (UB) for 60 min with all commercial kits used. The pretreatments tested in this communication were found to be adequate for next-generation sequencing (NGS) analysis, which will be part of an incoming original paper under construction.

## Data Availability

Not applicable.

## Supplementary Materials

The following supporting information can be downloaded at: https://www.mdpi.com/article/10.3390/ijms242015243/s1.
