# Supplementary material for: Physical Pretreatments Applied in Three Commercial Kits for the Extraction of High-Quality DNA from Activated Sewage Sludge
Source: Int J Mol Sci. 2023 Oct 17;24(20):15243. doi: 10.3390/ijms242015243 (PMC10607799; doi:10.3390/ijms242015243)
Supplement: Supplementary file 1 [file ijms-24-15243-s001.zip › ijms-2600571-supplementary.pdf]

Supplementary material:

Supplementary Table 1: Genomic DNA obtained with the extraction kit Soil (Nucleospin® Soil) from Macherey-Nagek. REF: 7407780.50 by default and the results obtained with the pre-treatments proven.

		Qubit concentration (ng µl^{-1})										
ID	Biomass (mg)	Default (kit)	80°C x 90 min	UB x 90 min	UB + 80°C x 30 min	UB + 80°C x 60 min	UB +80°C x 90 min	Average DNA concentration (ng µl^{-1})	Average biomass (mg)	Concentration (ng µl^{-1}) in 100 mg biomass	Quality 160/280	Average 260/280
1	166.1	0.178						0.249	194.2	0.128	1.642	1.522
2	228.2	0.318									1.506	
3	188.4	0.25									1.420	
4	194.7		13.7					12.800	163.4	0.732	1.617	1.647
5	152.7		14.4								1.664	
6	142.7		10.3								1.660	
7	164.5			0.346				0.361	162.9	0.222	1.846	1.848
8	170			0.448							1.833	
9	154.2			0.29							1.865	
10	144.7				0.878			1.009	137.8	0.732	1.617	1.647
11	132.9				1.1						1.664	
12	135.9				1.05						1.660	
13	146.5					21.8		22.933	143.8	15.952	1.826	1.831
14	150.9					22					1.820	
15	133.9					25					1.847	
16	138.6						31.6	22.733	146.4	15.525	1.558	1.550
17	117.3						14.6				1.468	
18	183.4						22				1.625	

Supplementary Table 2: Genomic DNA obtained with the extraction kit DNEasy® PowerSoil® from Qiagen. REF: 12888-100 by default and the results obtained with the pre-treatments proven.

		Qubit concentration (ng μl^{-1})										
ID	Biomass (mg)	Default (kit)	80°C x 90 min	UB x 90 min	UB + 80°C x 30 min	UB + 80°C x 60 min	UB +80°C x 90 min	Average DNA concentration (ng μl^{-1})	Average biomass (mg)	Concentration (ng μl^{-1}) in 100 mg biomass	Quality 160/280	Average 260/280
19	145	Not det.						0.000	166.0	0.000	1.368	1.475
20	155.4	Not det.									1.582	
21	197.5	Not det.									1.475	
22	178.2		15.2					14.433	138.3	10.436	1.827	1.858
23	135.5		14.9								1.871	
24	101.2		13.2								1.878	
25	113.5			Not det.				0.000	153.5	0.000	1.400	1.415
26	178.8			Not det.							1.301	
27	168.1			Not det.							1.545	
28	138.6				2.5			3.467	140.0	2.477	1.694	1.613
29	138.8				4.4						1.739	
30	142.5				3.5						1.408	
31	145.2					25.8		27.133	154.3	17.581	1.871	1.859
32	146.7					27.6					1.849	
33	171.1					28					1.858	
34	113.7						22.6	20.800	123.8	16.806	1.891	1.876
35	117.4						24.8				1.872	
36	140.2						15				1.865	

Supplementary Table 3: Genomic DNA obtained with the extraction kit E.Z.N.A.® Plant DNA Kit from Omega BIO-TEK. REF: D3485-01 by default and the results obtained with the pre-treatments proven.

		Qubit concentration (ng µl^{-1})										
ID	Biomass (mg)	Default (kit)	80°C x 90 min	UB x 90 min	UB + 80°C x 30 min	UB + 80°C x 60 min	UB +80°C x 90 min	Average DNA concentration (ng µl^{-1})	Average biomass (mg)	Concentration (ng µl^{-1}) in 100 mg biomass	Quality 160/280	Average 260/280
19	95.3	0.178						0.249	155.8	0.160	1.578	1.610
20	186	0.318									1.623	
21	186.2	0.25									1.628	
22	113.3		104					96.267	122.2	78.778	2.001	2.023
23	118.9		86								2.038	
24	134.4		98.8								2.030	
25	125			0.696				1.272	121.6	1.046	1.645	1.661
26	125.2			1.21							1.646	
27	114.5			1.91							1.692	
28	145.5				30.4			63.133	143.3	44.067	1.927	1.997
29	151.2				77.6						2.031	
30	133.1				81.4						2.033	
31	139.3					80.2		52.533	146.7	35.818	2.036	2.038
32	132.3					37.8					2.047	
33	168.4					39.6					2.032	
34	149.8						91.6	89.400	166.0	53.866	2.053	2.045
35	173.3						91				2.043	
36	174.8						85.6				2.040	
